# Changes in complement activation products after anti-VEGF injection for choroidal neovascularization in age-related macular degeneration and pachychoroid disease

**DOI:** 10.1038/s41598-021-87340-6

**Published:** 2021-04-19

**Authors:** Keiichiro Tanaka, Yasuharu Oguchi, Tomoko Omori, Yumi Ishida, Hiroaki Shintake, Ryutaro Tomita, Akihito Kasai, Masashi Ogasawara, Yukinori Sugano, Kanako Itagaki, Akira Ojima, Takeshi Machida, Hideharu Sekine, Tetsuju Sekiryu

**Affiliations:** 1 Department of Ophthalmology, Fukushima Medical University, Fukushima, 960-1295 Japan; 2 Department of Immunology, Fukushima Medical University, Fukushima, 960-1295 Japan

**Keywords:** Immunology, Medical research

## Abstract

We evaluated changes in the complement system resulting from anti-vascular endothelial growth factor (VEGF) in eyes with age-related choroidal neovascularization (CNV) including neovascular age-related macular degeneration, pachychoroid neovasculopathy, and polypoidal choroidal neovasculopathy. We measured the concentrations of the complement activation products (C3a, C4a), VEGF, and monocyte chemotactic protein-1 in the aqueous humor during intravitreal anti-VEGF injections for CNV. The VEGF level decreased significantly (*P* < 0.001), while the C3a and C4a levels increased significantly (*P* < 0.001 for both comparisons) 1 month after two monthly anti-VEGF injections. The VEGF level was correlated with the C3a (R = 0.328, *P* = 0.007) and C4a (R = − 0.237, *P* = 0.055) levels at baseline, but the correlation between the VEGF and C3a levels (R = − 0.148, *P* = 0.242) changed significantly (*P* = 0.028 by analysis of covariance) after anti-VEGF treatment. The C3a increase after anti-VEGF therapy did not change the visual outcomes in eyes with CNV for 1 year. Dysregulation of the complement system can be induced after anti-VEGF therapy.

Age-related macular degeneration (AMD) is a leading cause of blindness in developed countries^1^.The estimated number of people with AMD is 196 million in 2020 and is expected to increase to 288 million in 2040 worldwide^2^. Therefore, an effective therapeutic strategy for AMD treatment has a significant impact on elderly persons and society as a whole. AMD is classified as atrophic or neovascular AMD; the former is characterized by atrophy of the photoreceptors and retinal pigment epithelium (RPE) without exudative changes and the latter by choroidal neovascular membranes (CNV) and retinal edema at the macula. Recently, “pachychoroid disease” that includes pachychoroid neovascularization (PNV) and polypoidal choroidal vasculopathy (PCV) was proposed. PNV and PCV are characterized by type 1 CNV with a thick choroid and no or rare soft drusen^3^.

Although the AMD etiology is unknown, a disorder of the complement system may be deeply involved in AMD development. Previous genetic studies have reported the high incidence of the polymorphism related to the complement system in patients with AMD^4^. Histopathologic studies reported that complement activation products deposit in the drusen on the RPE cells, Bruch’s membrane, and choriocapillaris^5,6^.

The complement system plays a pivotal role both in the innate and adaptive immune system. The complement system is comprised of three activation pathways: classical, lectin, and alternative pathways. Complement activation finally leads to phagocytosis and the cell-killing process by the membrane attack complex. Complement activation products 3a (C3a), 4a (C4a), and 5a (C5a) are fragments resulting from complement activation, which cause smooth muscle contraction, vasodilation, histamine release from mast cells, and enhance vascular permeability^7^. Activation of all three pathways generate C3a. C4a is induced in the classical and lectin pathway. C5a is generated as a result of activation of the terminal pathway. Elevated C3a in the intraocular fluid in neovascular AMD (nAMD) suggests the involvement of the complement system^8^.

Proangiogenic cytokines are also profoundly involved in the development of nAMD. Anti-vascular endothelial growth factor (VEGF) drugs have revolutionized treatment of nAMD. Intravitreal administration of anti-VEGF drugs has become the first-line treatment. However, recent reports have enumerated several concerns about anti-VEGF therapy.

RPE atrophy developed in 10% to 20% of patients treated with anti-VEGF therapy during 5 years of follow-up^9^. Inflammation, photooxidation of retinol derivatives^5^, oxidative stress^10^, and lipid accumulation^11^ may induce RPE degeneration resulting in loss of photoreceptors and choriocapillaris^12^. The photoreceptors, RPE, and choriocapillaris complex survive these cellular stresses by organizing the secretory protein network^13^. VEGF is a key cytokine in this network. Reduced VEGF secretion from the RPE by paracrine and autocrine can cause atrophy of the RPE and endothelium of the choriocapillaris. The number of fenestrations in the endothelial cells of the choriocapillaris decreases after intravitreal bevacizumab (Avastin, Genentech Inc., South San Francisco, CA, USA) injections in primates, and the elimination of VEGF-induced degeneration of the photoreceptors and choriocapillaris in mice^14^. Considering these facts, some researchers have pointed out that anti-VEGF administration could lead to RPE atrophy^14–16^. However, the mechanism of RPE atrophy after anti-VEGF therapy is not well understood. A genetic analysis recently suggested that the polymorphism of the gene encoding complement factor H (CFH) reduced the response to anti-VEGF therapy^17^. An interaction between anti-VEGF therapy and the complement system may be associated with the ocular tissue damage after anti-VEGF treatment.

The cytokines including VEGF and complement activation products are measurable in the aqueous humor of eyes with nAMD^8,18^. Analysis of the aqueous humor can help investigate the interaction between VEGF and complement systems after intravitreal anti-VEGF injections. The results of previous studies regarding complement activation in the aqueous humor after anti-VEGF therapy were controversial. Keir et al. reported elevated C3a after intravitreal bevacizumab injections in small number of patients with nAMD^19^. Schick et al. reported that the C3a concentrations in the aqueous humor were not elevated in the patients including undergoing anti-VEGF treatment^8^.

To clarify the changes in complement activation products after anti-VEGF therapy in a clinical setting, we measured the concentrations of C3a, C4a, and proangiogenic cytokines, VEGF, and monocyte chemoattractant protein-1 (MCP-1) in the aqueous humor at baseline and  one months after two monthly intravitreal injections in eyes with CNV in nAMD, PNV, and PCV. The measured protein levels were compared to the 12-month treatment outcomes of anti-VEGF therapy.

## Results

### Clinical features

Table 1 shows the demographic and clinical characteristics of patients with CNV and controls. A total of 102 patients (eyes) (72 patients with CNV and 30 controls) were enrolled. To avoid ambiguity or confusion, we use the words drusen-associated nAMD to indicate typical nAMD, which is common in Caucasian patients. The patients with drusen-associated nAMD were a median age of 73.0 years (interquartile range [IQR], 68.0–78.0) and the controls were a median age of 69.0 years (IQR 67.0–74.5) (*P* = 0.118). The proportion of female patients with CNV (n = 20) was lower than that in the control group (n = 17, *P* = 0.006). Of the 72 eyes with CNV, the numbers of eyes with PNV, PCV, and drusen-associated nAMD were 22 (30.6%), 25 (34.7%), and 25 (34.7%), respectively. There were no significant differences in age (*P* = 0.054), sex (*P* = 0.218), logarithm of the minimum angle of resolution (logMAR) (*P* = 0.212), axial length (*P* = 0.902), equivalent spherical power (*P* = 0.166), incidence of subretinal fluid (SRF) (*P* = 0.06), intraretinal fluid (IRF) (*P* = 0.288), pigmentary abnormalities (*P* = 0.501), greatest linear dimension (GLD) (*P* = 0.916), CNV lesion size (*P* = 0.146), and central retinal thickness (CRT) (*P* = 0.349) among the three subtypes. The incidence rates of soft drusen and subfoveal choroidal thickness (SFCT) (*P* < 0.001 for both comparisons) differed among the three subtypes, with the SFCT thicker in the PNV (*P* < 0.001) and PCV groups (*P* = 0.004) than in the drusen-associated nAMD group. There was no difference in the SFCT between the PNV and PCV (*P* = 0.385) groups.

**Table 1:** Demographics of patients with CNV and controls ar baseline

	Eyes with CNV (n = 72)					Controls (n = 30)	P**
	PNV (n = 22)	PCV (n = 25)	DA nAMD (n = 25)	P*	All eyes with CNV (n = 72)		
Age, median (IQR) (year)	72.0 (63.8–76.3)	72.0 (66.5–77.5)	77.0 (69.0–82.0)	0.054	73.0 (68.0–78.0)	69.0 (67.0–74.5)	0.118
Female, sex, n (%)	4 (18.2)	6 (24.0)	10 (40.0)	0.218	20 (27.8)	17 (56.7)	0.006
LogMAR, median (IQR)	0.22 (0.05–0.43)	0.22 (0.10–0.35)	0.22 (0.15–0.70)	0.212	0.22 (0.10–0.52)	0.35 (0.22–0.57)	0.020
Axial length, median (IQR) (D)	23.58 (23.04–23.88)	23.55 (22.91–24.25)	23.51 (22.93–24.21)	0.902	23.57 (22.97–24.18)	24.04 (23.36–24.38)	0.040
Spherical equivalent, median (IQR) (D)	0.50 (− 0.63 to 2.50)	0.25 (− 1.44 to 1.31)	− 0.63 (− 2.13 to 1.25)	0.166	0.00 (− 1.36 to 1.44)	− 0.31 (− 2.47 to 0.25)	0.050
CRT, median (IQR) (μm)	404 (213–551)	392 (271–510)	422 (348–575)	0.349	402 (288–548)	–	–
SFCT, median (IQR) (μm)	344 (261–415)	268 (191–356)	182 (107–252)	< 0.001	260 (170–358)	–	–
GLD, median (IQR) (μm)	3.96 (2.92–6.29)	3.89 (3.52–6.05)	4.55 (2.69–6.58)	0.916	4.19 (3.09–6.24)	–	–
CNV size, median (IQR) (mm^2^)	1.50 (0.90–4.09)	1.06 (0.64–1.22)	1.27 (0.39–2.70)	0.146	1.18 (0.66–2.15)	–	–
SRF, n (%)	19 (86.4)	24 (96.0)	18 (72.0)	0.060	61 (84.7)	–	–
IRF, n (%)	4 (18.2)	5 (20.0)	9 (36.0)	0.288	18 (25.0)	–	–
Soft drusen, n (%)	0 (0)	0 (0)	16 (64.0)	< 0.001	16 (22.2)	–	–
Pigmentary abnormality, n (%)	12 (80.0)	17 (89.5)	15 (75.0)	0.501	44 (81.5)		

The Kruskal–Wallis test was used for comparisons between three categories. The Mann–Whitney U test was used for comparisons between all eyes with CNV patients and controls.

nAMD, neovascular age-related macular degeneration; PNV, pachychoroid neovasculopathy; PCV, polypoidal choroidal vasculopathy; DA nAMD, drusen-associated neovascular age-related macular degeneration; SD, standard deviation; PVD, posterior vitreous detachment; CRT, central retinal thickness; SFCT, subfoveal choroidal thickness; GLD, greatest linear dimension; CNV, choroidal neovascularization; SRF, subretinal fluid; IRF, intraretinal fluid.

*The Kruskal–Wallis test was used for analyses among the nAMD subtypes.

**The Mann–Whitney U-test was used for analyses between all nAMD and controls.

### Levels of complement activation products and cytokines

No patients had clinically significant inflammation after intravitreal aflibercept (Eylea, Regeneron Pharmaceutics, Tarrytown, NY, USA) injections (IAIs) in our series. The median levels of C3a, C4a, VEGF, and MCP-1 in the aqueous humor were 2480 (IQR 1862–3433) pg/mL, 1545 (IQR 1146–1919) pg/mL, 36.68 (IQR 20.90–51.50) pg/mL, and 252.6 (IQR 178.8–382.8) pg/mL, respectively, in the eyes with CNV, and 1929 (IQR 1374–3010) pg/mL, 1278 (IQR 836–1638) pg/mL, 23.79 (IQR 16.34–35.11) pg/mL, and 174.8 (IQR 116.1–260.3) pg/mL in the controls at baseline (Table 2).

**Table 2:** Changes in Levels of complement factors and cytokines in the aqueous humor-patients and controls.

	Controls (n = 30)	*P* (Controls vs Baseline)	Eyes with CNV (n = 72)		
			Baseline	2 months	*P* (Baseline vs 2 months)
C3a, median (IQR) (pg/mL)	1929 (1374–3010)	0.033	2480 (1862–3433)	3095 (2524–4464)	< 0.001
C4a, median (IQR) (pg/mL)	1278 (836–1638)	0.021	1545 (1146–1919)	1633 (1353–2153)	< 0.001
VEGF, median (IQR) (pg/mL)	23.79 (16.34–35.11)	0.016	36.68 (20.90–51.50)	8.57 (4.31–18.29)	< 0.001
MCP1, median (IQR) (pg/mL)	174.8 (116.1–260.3)	0.002	252.6 (178.8–382.8)	262.4 (193.1–331.3)	0.570

The data were analyzed using the Mann–Whitney U-test; the eyes with CNV and controls, and baseline and at 2 months. C3a, complement component 3a; C4a, complement component 4a; VEGF, vascular endothelial growth factor; MCP-1, macrophage chemoattractant protein 1. IQR, interquartile range.

In the eyes with CNV,  one month after two monthly injections, the median C3a levels increased to 3095 (2524–4464) pg/mL at 2 months, and the median C4a levels increased to 1633 (1353–2153) pg/mL (*P* < 0.001 for both comparisons). While the median VEGF levels decreased to 8.57 (4.31–18.29) pg/mL from 36.68 (20.90–51.50) (*P* < 0.0001), the VEGF level in 15 of 72 eyes (20.8%) was under the limit of detection (LOD) 2 months after the treatment. No significant differences were seen in the median MCP-1 (*P* = 0.57) levels between baseline and 2 months later.

The tendency of VEGF to decrease at 2 months was similar in each subgroup. The C3a level in PNV (*P* = 0.017) and drusen-associated nAMD (*P* < 0.001) increased 2 months after the initial treatment. The MCP-1 level in PCV (*P* = 0.023) decreased and drusen-associated nAMD (*P* = 0.045) increased 2 months after the initial treatment (Table 3).

**Table 3:** Changes in Levels of complement factors and cytokines in the aqueous humor of patients with CNV.

Analyte median (IQR) (pg/mL)	PNV (n = 22)			PCV (n = 25)			DA nAMD (n = 25)		
	Baseline	2 months	*P*	Baseline	2 months	*P*	Baseline	2 months	*P*
C3a	2059 (1853–3171)	3406 (2194–4760)	0.017	2668 (1886–3932)	3084 (2701–4956)	0.187	2539 (1811–3319)	3047 (2453–3748)	< 0.001
C4a	1518 (971–2066)	1683 (1494–1889)	0.003	1601 (1192–2133)	1762 (1252–2546)	0.804	1503 (1147–1832)	1594 (1231–2184)	< 0.001
VEGF	35.67 (25.03–47.27)	08.39 (6.34–16.32)	< 0.001	38.73 (20.47–65.93)	12.90 (04.31–22.44)	< 0.001	36.07 (18.45–52.21)	6.96 (4.06–17.61)	0.011
MCP1	223.8 (131.1–386.8)	203.0 (117.3–315.8)	0.649	314.2 (231.4–476.9)	265.1 (192.9–327.9)	0.023	266.6 (179.1–291.4)	289.1 (226.1–344.1)	0.045

Changes in levels of complement factors and cytokines in the aqueous humor of eyes with choroidal neovascularization (CNV). Wilcoxon signed rank test was used for comparisons between baseline and 2 months.

nAMD, neovascular age-related macular degeneration; PNV, pachychoroid neovasculopathy; PCV, polypoidal choroidal vasculopathy; DA nAMD, drusen associated neovascular age-related macular degeneration; IQR, interquartile range; C3a, complement component 3a; C4a, complement component 4a; VEGF, vascular endothelial growth factor; MCP-1, macrophage chemoattractant protein 1. The Mann–Whitney U-test was used for analysis between baseline and 2 months.

**Figure 1:**
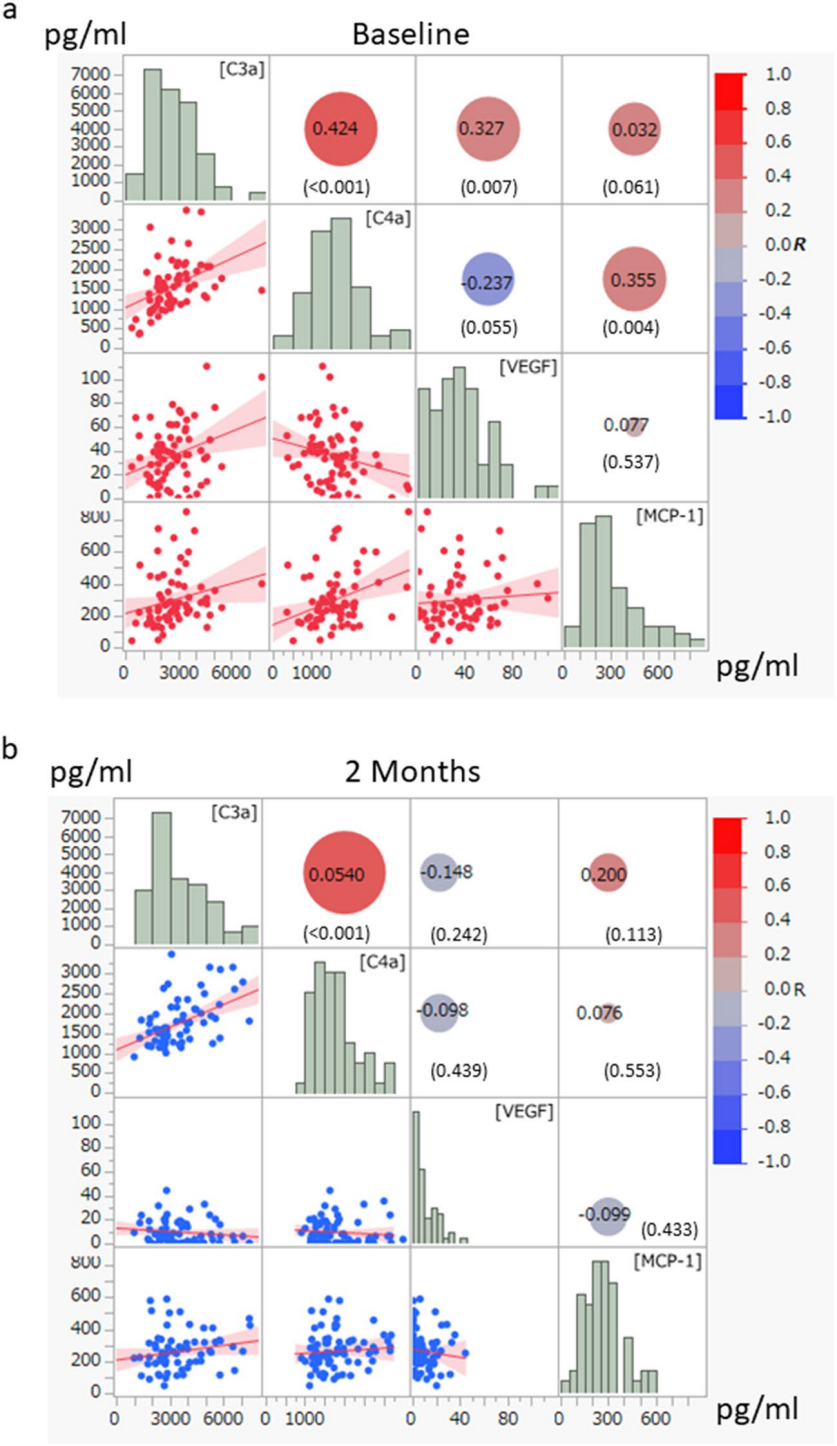
The correlation among complement factors and cytokines in the aqueous humor of eyes with choroidal neovascularization (CNV). Pearson’s correlation analysis was used. C3a, complement component 3a; C4a, complement component 4a; VEGF, vascular endothelial growth factor; MCP-1, macrophage chemoattractant protein 1. R, correlation coefficient. The scale of the axis is adjusted in the same range between (**a**) and (**b**). the sizes of the circles indicate the magnitude of the correlation; red circle, positive correlation; blue circle, negative correlation. Number in parenthesis indicates the *P* value of R.

**Figure 2:**
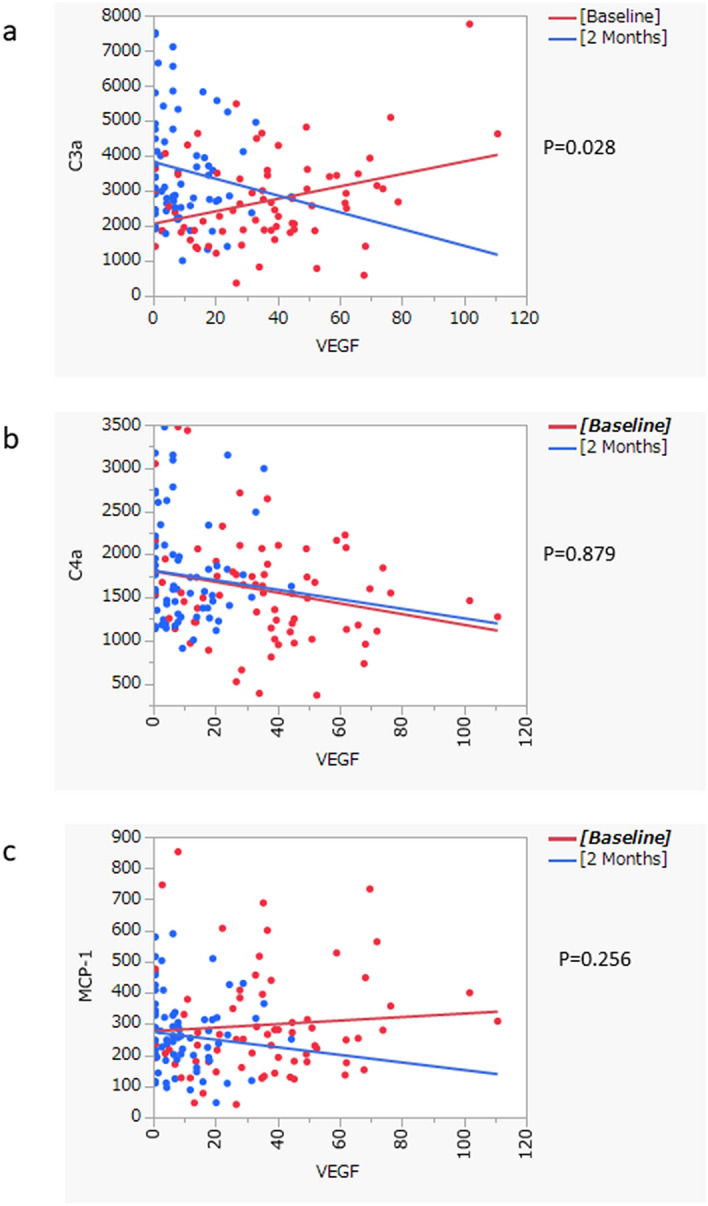
Analyses of covariance. Red dot: baseline. Blue dot: 2 months. The correlation between VEGF and C3a levels (**a**) is statistically different between baseline and 2 months (*P* = 0.028). There was no difference in C4a (**b**) and MCP-1 levels (**c**).

The C3a level was correlated positively with the VEGF level at baseline (R = 0.327, *P* = 0.007), whereas it was correlated negatively with the C3a level at 2 months after IAI (R = − 0.148, *P* = 0.242) (Fig. 1). The C4a level was correlated negatively with the VEGF level both at baseline (R = − 0.237, *P* = 0.055) and 2 months (R = − 0.098, *P* = 0.439) after treatment. The MCP-1 level was correlated positively with the VEGF level at baseline (R = 0.537, *P* = 0.077) and negatively at 2 months (R = − 0.099, *P* = 0.433). The correlations between the C3a, C4a, MCP-1, and VEGF levels were analyzed by analysis of covariance. The correlation between the C3a and VEGF levels differed significantly at baseline and 2 months (*P* = 0.028) (Fig. 2). The C3a level was especially higher in the eyes with VEGF under the LOD.

The influence of the C3a level on the outcome of anti-VEGF treatment was analyzed in a limited number of the patients (37 patients). The C3a levels at baseline and 2 months were not correlated with the changes in the logMAR VA at 12 months and the number of injections administered over 12 months (Table 4). Dry maculas (defined as those with no SRF, IRF, or hemorrhages) were achieved at 12 months in 22 (59%) of 37 eyes. The median C3a levels in the eyes with dry maculas at 12 months were 2654 pg/mL (IQR 2167–3338, *P* = 0.687**)** at baseline and 2727 pg/mL at 2 months (IQR 2332–3144; *P* = 0.621).

**Table 4:** C3a and treatment outcomes at twelve months.

Analyte	C3a at baseline		C3a at 2 months	
		*P* value		*P* value
logMAR change	rs = − 0.0744	0.662	rs = − 0.2006	0.234
Number of injections	rs = 0.0806	0.635	rs = 0.0864	0.611
Dry macula (22 of 37 eyes) median (IQR)	2654 pg/mL (2167–3338)	0.687	2727 pg/mL (2332–3144)	0.621

rs: correlation of Spearman’s analysis.

C3a, complement component 3a; VEGF, vascular endothelial growth factor.

## Methods

### Patients

This prospective observational study adhered to the tenets of the Declaration of Helsinki. The Institutional Review Board of Fukushima Medical University approved this research before patient enrollment began at the Department of Ophthalmology, Fukushima Medical University Hospital, Fukushima City, Japan. All patients provided informed consent before enrollment.

Seventy-two treatment-naïve eyes of patients over 50 years old and age-matched 30 eyes were included. All patients underwent comprehensive ophthalmic examinations; slit-lamp biomicroscopy with non-contact fundus lens, and funduscopy. Color fundus photographs and fluorescein angiography (FA) were obtained using a fundus camera TRC-DX™ (Topcon, Tokyo, Japan). Fundus autofluorescence and indocyanine green angiography (ICGA) images were obtained using the HRA2™ (Heidelberg Engineering, Heidelberg, Germany). Optical coherence tomography (OCT) (Spectralis™, Heidelberg Engineering) and OCT angiography (PLEX Elite 9000™, Carl Zeiss Meditec, Inc., Dublin, CA, USA) also were performed.

Seventy-two eyes of elderly patients with CNV included PNV and PCV. The differences in the pathogenesis among PNV, PCV, and typical nAMD, namely drusen-associated nAMD, continue to be discussed. However, the clinical features of CNV in elderly persons are common characteristics in three categories. We used drusen-associated nAMD to indicate typical nAMD, the clinical features of which were defined as nAMD characterized by soft drusen exceeding 63 microns or a total area exceeding a 125-μm circle^20^ in the treated eyes or contralateral eyes, excluding PNV, PCV, and retinal angiomatous proliferation. The diagnoses of PNV and PCV were made based on previous reports^3,21,22^. The diagnostic criteria for PNV were type 1 CNV detected in one or both eyes; no or non-extensive drusen (total area ≤ 125-μm circle), or hard drusen (≤ 63 μm) in both eyes (Age-Related Eye Disease Study^20^: category 1, no AMD), choroidal vascular hyperpermeability detected on late-phase ICGA images; dilated choroidal vessels below type 1 CNV detected by ICGA and OCT; and the presence of central serous chorioretinopathy or pachychoroid pigment epitheliopathy-related RPE abnormalities independent of CNV lesions detected by fundus autofluorescence or a history of central serous chorioretinopathy. PCV was diagnosed in the presence of a branching vascular network with terminal aneurysmal dilatations on ICGA images corresponding to a steep RPE elevation seen on OCT images. Other characteristics on the OCT, FA, and ICGA images were the same as PCV. The exclusion criteria were eyes with a history of any other retinal diseases; uveitis; glaucoma including ocular hypertension with any antiglaucoma eye drops; any intraocular surgery including cataract surgery; or high myopia (spherical equivalent <  − 6 diopters or axial length > 26.5 mm), or eyes having received intravitreal anti-VEGF injections in the past; and patients with any systemic diseases potentially involved in complement system activation, such as diabetes, autoimmune diseases, cancer, cardiovascular disease, cerebrovascular disease, or systemic corticosteroid medications. The aqueous humor of patients with cataracts without drusen or retinal disease on funduscopy and OCT was measured as the control group.

### Image analysis

Soft drusen and pigmentary abnormalities were evaluated using color fundus photographs. Soft drusen were graded based on the simplified severity scale for AMD from the Age-Related Eye Disease Study^20^. The CRT, SRF, IRF, and SFCT were evaluated on OCT images. The CRT and SFCT were measured using the caliper function of the Spectralis™. The CRT was defined as the distance between the surface of the RPE and that of the internal limiting membrane. SFCT was defined as the distance between the hyperreflective line corresponding to the Bruch’s membrane beneath the RPE and the chorioscleral border. The diagnosis of polypoidal lesions was confirmed by the presence of dilated polyps at the end of the branching vascular network on ICGA images. Choroidal vascular hyperpermeability was defined as multifocal hyperfluorescence in the middle and late phases of ICGA^3^. The GLD was measured manually using a tool in the software of the Topcon ImageNet system as the lesion dimension covering the CNV area, including the areas of dye leakage, pigment epithelial detachment, and subretinal hemorrhage on FA. The CNV lesion size was calculated manually in each early ICGA image using the draw lesion tool in the Image explorer attached to the HRA2.

### Treatment regimen

All patients initially received a monthly IAI (2 mg) injection three times as a loading dose at the beginning of the treatment and 1 and 2 months later; IAI was administered based on a treat-and-extend regimen. In that regimen, IAI prolonged the administration interval by 1 month if there were no exudative changes or hemorrhage in the macula, and shortened the injection interval by 1 month if a subretinal hemorrhage, retinal edema, or serous retinal detachment was present. The maximal dosing interval was 3 months.

### Aqueous humor collection

Aqueous humor was aspirated before IAI and cataract surgery under topical anesthesia using a syringe with a 30-gauge needle (Nipro, Osaka, Japan) by the same procedure. The aqueous humor samples were immediately mixed with 2 μL of protease inhibitor cocktail (Sigma, St. Louis, MO, USA) to prevent complement and anaphylatoxin activation. Aliquots of the obtained aqueous humor samples were stored at − 80 °C until analysis.

### Measurements of complement activation products and cytokines levels

The levels of complement activation products (C3a and C4a) in the aqueous humor were measured using an Human Anaphylatoxin Kit (BD Biosciences, Franklin Lakes, NJ, USA). The levels of cytokines (VEGF and MCP-1) in the aqueous humor were measured using a Human Soluble Protein Master Buffer Kit (BD Biosciences). The bead-based immunoassay was performed according to the manufacturer’s instructions.

### Statistical analysis

The Mann–Whitney U-test was used to compare the levels of complement activation products and cytokines in the aqueous humor between the groups. The Kruskal–Wallis test was used to compare the levels of anaphylatoxins and cytokines in the aqueous humor among the three CNV subtypes. Post-hoc analysis was performed by Steel–Dwass analysis. The differences in the cytokine and C3a and C4a levels between baseline and 2 months were calculated using the Wilcoxon signed-rank test. The Pearson correlation coefficient was used to evaluate the correlation between each measurement. The evaluation of the linear regression in two groups was performed by analysis of covariance. When we evaluated the correlation between the VEGF level after anti-VEGF treatment and another measurement, the VEGF level under the LOD was substituted by the minimal value of the VEGF. The correlation between the C3a levels, logMAR changes, and number of injections was analyzed by Spearman’s rank correlation analysis. Statistical analyses were performed using JMP software (SAS Institute Inc, Cary, NC, USA). *P* < 0.05 was considered statistically significant.

## Discussion

We measured the changes in the complement activation products and cytokines in the aqueous humor of eyes with drusen-associated nAMD, PNV, and PCV during anti-VEGF therapy. The VEGF level decreased significantly, while the C3a level increased at 2 months after the initial anti-VEGF injections. The tendency of C3a to increase and for VEGF to decrease at 2 months was similar in each CNV category. The correlations between the C3a and VEGF levels differed at baseline and 2 months. The activation of the complement system after anti-VEGF therapy did not change the clinical outcome of the eyes with CNV for 1 year.

Anti-VEGF therapy is a remarkable advancement in nAMD treatment and has become a current therapeutic standard. The decade of experience with anti-VEGF therapy for nAMD has shed light on the undesirable consequences of this therapy. Long-term treatment can reduce response to anti-VEGF drugs^23,24^ and RPE atrophy^15,16^. The mechanism of ocular tissue damage by anti-VEGF therapy has not been elucidated. Inflammation after anti-VEGF therapy was suggested as a causative factor of the tissue damage^25,26^.

The current study showed that the C3a level increased at 2 months after anti-VEGF treatment, while the VEGF levels decreased significantly. These results were consistent with a previous study. Keir et al. reported that C3a was elevated in the aqueous humor 48 h after intravitreal bevacizumab injections in 10 patients with nAMD^19^. The investigators found that CFH decreased after anti-VEGF treatment in an experimental study. The authors hypothesized that increasing the vulnerability of complement activation by decreasing CFH after anti-VEGF injection may increase the C3a level. We found that the C3a elevation continued for 1 month after anti-VEGF injection in the clinical setting. However, there is still room for discussion because the positive correlation between the C3a and VEGF levels changed after anti-VEGF treatment. We should consider two possibilities. One is that C3a generation is, indeed, not controlled by VEGF. Another possibility is that residual anti-VEGF drugs that combine with VEGF could mask the VEGF elevation. Therefore, this possibility implies VEGF involvement in C3a generation. The interaction between the complement system and VEGF warrants further investigation.

A difference in the pathophysiology between PNV and drusen-associated nAMD was suggested. A hypoxia-driven mechanism in PNV was postulated from an OCT angiography study^27^. However, the correlation between VEGF and C3a after anti-VEGF therapy was similar among PNV, PCV, and drusen-associated nAMD. The changes after anti-VEGF injection may be common regardless of the pathophysiology of the neovascularization.

Elevated C3a can damage the choroidal vasculature. A recent study reported that the choriocapillaris expresses the cell cycle gene that responds to complement activation and induces apoptosis of endothelial cells^28^.The expression of this gene was examined along the choroidal vessel tree. The endothelial cells of the choriocapillaris from patients with nAMD specifically expressed this gene compared to arterial and venous endothelial cells. If the endothelial cells of the choriocapillaris are exposed to complement activation products by repeated treatments with anti-VEGF drugs, the function and number of endothelial cells may decrease, causing circulatory insufficiency of the choriocapillaris and RPE atrophy. Interestingly, Sakamoto et al.^26^ reported that only matrix metalloproteinase-9 (MMP9) was the cytokine that increased after anti-VEGF injections among several cytokines 2 months after anti-VEGF injections. MMP9 reportedly increases when the endothelial cells are damaged by the membrane attack comples^29^, indicating complement-induced cell injury. Together with increasing intraocular C3a, the vascular endothelial cells may be damaged by the complement system after anti-VEGF injections.

The choroidal thickness decreases as a result of IAI with a treat-and-extend regimen for nAMD, especially in PNV and PCV^30^. Thus far, the choroid might become thinner as a result of anti-VEGF treatment during recovery of the choroidal vasculature from the pathologic changes in nAMD. However, we reported that the tendency for the choroid to become thin did not stop during 3 years of anti-VEGF therapy^31^. The VEGF levels after the treatment reportedly decreased below the lower limit of the quantification for about 3 months maximally^32^. The long-term complement dysregulation by low VEGF may affect the choroidal vasculature resulting in long-lasting choroidal thinning after the treatment.

The C4a level was higher in the eyes with CNV and elevated after anti-VEGF therapy in our study. The activation of the classical or lectin pathways in nAMD was reported previously^33^. Regarding C4a elevation after the injection, aflibercept is potentially associated with the activation in the classical pathway. Bevacizumab is a humanized monoclonal IgG1 antibody which potentially causes intraocular inflammation after the injection^34^. IgG1 antibody is composed of Fab and Fc domain. The classical pathway was reportedly activated by the Fc portion of the antibody^35^. Aflibercept is a recombinant fusion glycoprotein comprised of the second immunoglobulin-like C2 domain of human VEGF receptor (VEGFR) 1, the third Ig-like C2 domain of human VEGFR 2, and the Fc domain of human IgG1. Degraded aflibercept could be associated with the increase in C4a after the injection.

Noninfectious inflammation was reported after anti-VEGF injections^34^. Although the incidence was very low, most anti-VEGF agents have caused inflammation after intravitreal injections^36–38^. Complement dysregulation may be a predisposing factor after the anti-VEGF treatment.

The complement system activation after anti-VEGF therapy may affect the clinical outcomes of anti-VEGF therapy. We did not find a significant difference between the elevation of C3a after anti-VEGF treatment and the logMAR changes, achievement of dry maculas, and the number of injections for 12 months after treatment. These undesirable responses could not reflect clinical outcomes since VEGF production may recover at some time regardless of whether it is pathologic or not. However, long-term unnecessary complement activation by anti-VEGF therapy can reduce the efficacy of anti-VEGF drugs^23^ or induce tissue damage by local depletion of VEGF^15,16^. Consideration should be given to overtreatment when choosing the treatment regimen to avoid the undesirable consequences of anti-VEGF therapy.

The current study had a few limitations First, there may be a selection bias regarding patients because this study was performed in one institution. Second, the consistency of the PNV diagnosis was insufficient because the criteria are not established. The diagnosis of PNV was based on previous reports^3,21,39^. Third, the VEGF concentration after anti-VEGF drugs may have been underestimated, as suggested by Takahashi et al.^40^. Although the measured value of the VEGF concentration itself may be low, its correlation with C3a can be evaluated. Fourth, C5a was not evaluated because the measurements of C5a in the aqueous humor were out of range in some samples and should be evaluated in another study. Fifth, we recruited the eyes with cataract as controls, and these eyes could be associated with intraocular inflammation. However, it is ethically difficult to collect aqueous humor from completely healthy eyes. The healthy eyes without cataracts should be examined in a future study.

Although this study suggested the interaction between VEGF and the complement system, we could not fully elucidate the mechanism of the interaction. Further studies are needed.

In conclusion, we demonstrated dysregulation of the complement system following anti-VEGF therapy for nAMD. Physicians should consider treatment regimens to avoid overtreatment during prophylactic or intensified treatment for nAMD.
